# Two inflammasomes as tumour markers for nonmelanocytic skin cancer: new hopes for early detection and targeted therapy

**DOI:** 10.1093/skinhd/vzaf106

**Published:** 2026-04-20

**Authors:** Azadeh Khayyat, Seyed Amir Zohouri, Mohammad Ali Esmaeil Pour, Parvaneh Hatami, Bruce R Smoller

**Affiliations:** Pathology Department, Medical College of Wisconsin, Milwaukee, WI, USA; Simon Fraser University, Burnaby, BC, Canada; Rheumatology Department, Medical College of Georgia, Augusta, GA, USA; Dermatology Department, Tehran University of Medical Sciences, Tehran, Iran; Dermatopathology Department, University of Rochester Medical Center, Rochester, NY, USA

## Abstract

Nonmelanoma skin cancer (NMSC) refers to the most common skin cancers, including basal cell carcinoma (BCC) and squamous cell carcinoma (SCC). Therefore, dependable biomarkers could benefit early predictive diagnosis and become therapy targets. Recent findings have implicated the role of inflammasomes, including (NLRP1 and NLRP3), in skin carcinogenesis. This literature review shall further examine and summarize whether NLRP1 and NLRP3 inflammasomes could be potential tumour markers of NMSC. The literature in this area is synthesized, with the aim of providing a broad understanding of the issue concerning skin cancer. A complete literature search of PubMed, Google Scholar and Web of Science was conducted including publications covering the period January 2000–May 2024. Fifteen relevant articles were included and helped to shed light on the roles of NLRP1 and NLRP3 in NMSC. The included articles comprised original research articles and review papers. In the context of tumour regression and progression, low expression of the NLRP1 inflammasome indicated an increase in metastasis and recurrence in patients with NMSC. Skin cancer is facilitated by a germline mutation in *NLRP1*. NLRP1 inflammasomes have become a focal point in skin biology as mutations in *NLRP1* contribute to the genetic basis of dermatological diseases and heighten the risk of skin cancer. The role of the NLRP3 inflammasome, particularly activation associated with chronic inflammation and cancer progression, involves increased cytokine production and creation of a protumorigenic environment. The NLRP1 and NLRP3 inflammasomes are potential biomarkers for early detection, prognosis and therapeutic targets. Targeting inflammasomes could be a crucial strategy in alleviating skin inflammation, combating photoageing and decreasing the risk of epithelial skin tumour development. NLRP1 and NLRP3 inflammasomes are promising tumour markers for NMSC. A role in inflammation or tumour growth and metastasis provides a good rationale for early detection and prognosis, and targeted clinical applications. This study identifies NLRP1 and NLRP3 inflammasomes as potential biomarkers and therapeutic targets in NMSC, linking their altered expression to tumour progression and recurrence.

## Teaser

Our literature review comprehensively examines the roles of NLRP1 and NLRP3 inflammasomes as tumour markers for non-melanoma skin cancer (NMSC). We have collated and analysed data from various studies to provide a thorough understanding of how these inflammasomes contribute to the pathogenesis and progression of NMSC.

Nonmelanoma skin cancers (NMSCs) include mainly basal cell carcinoma (BCC) and squamous cell carcinoma (SCC). They represent the most common types of skin cancers found worldwide. Although they do not share the same life-threatening reputation as melanomas, their potential for high recurrence rates and ability to metastasize significantly affects the health of patients. As the incidence of NMSCs is rising worldwide, it is becoming more important to find robust markers for early diagnosis and prognosis, as well as to develop effective treatment. More recent studies^1,2^ have shifted attention to the participation of NOD-like receptor family, pyrin domain containing 1 (NLRP1) and NOD-like receptor family, pyrin domain containing 3 (NLRP3) inflammasomes in the development of skin cancers, and have highlighted them as promising new markers and therapeutic targets in NMSCs. Inflammasomes are protein complexes that, by recognizing danger signals, activate the innate immune response via the secretion of inflammatory molecules such as interleukin (IL)-1β and IL-18. They may exert diverse effects. On the one hand, they contribute to the body’s defence against infections and have a role in tissue repair; on the other hand, when the inflammatory response becomes dysregulated, they might predispose to cancer. Inflammasomes, such as NLRP1 and NLRP3, have been implicated in various cutaneous diseases, from benign to malignant conditions. Activation of such inflammasomes may lead to a unique form of cell death known as pyroptosis, which may promote inflammation but, paradoxically, favour tumour development. Such duplicity testifies to how the inflammasomes are involved in the most intricate parts of skin cancer biology.^3,4^ Expression of NLRP1 and NLRP3 in different tissues may alter their role in carcinogenesis. NLRP1 is more commonly expressed by T cells and Langerhans cells, which are involved in cutaneous immune defence; however, expression of NLRP3 is limited and only seen in selected tissues, including the oesophagus, bladder and oropharynx.^4^ This unique tissue distribution highlights how these inflammasomes can differently affect immune responses and NMSC, depending on their site of action in the body. Genetic mutations in *NLRP1* have been associated with certain inflammatory syndromes and enhanced cancer risk, rendering it a potential focal point for skin cancer research.^2^

More recent studies have started to unveil how the activation of *NLRP1* and *NLRP3* can affect the tumour environment by modulating mainly inflammation and cancer cell behaviour. For example, reduced expression of *NLRP1* has been associated with metastasis and recurrence in patients with NMSC, indicating that it could one day become a useful marker for determining prognosis.^1^ However, chronic activation of *NLRP3* has been linked to sustained inflammation, predisposing to many cancers. Inflammasomes can support relentless cell proliferation and resistance to cell death through genetic and epigenetic changes important for tumorigenesis.^3,5^

Excitingly, targeting *NLRP1* and *NLRP3* may open up new therapeutic avenues. Increasing evidence from preclinical studies has shown that selective inhibition of *NLRP3* may reduce inflammation and slow tumour growth, suggesting potential treatment strategies for NMSC.^6^ This creates a window for designing more specific therapies that could reduce side effects by targeting specific inflammasome pathways, considering that NLRP1 and NLRP3 have tissue-specific roles. With continued research, these findings are hoped to offer conclusive evidence of the use of NLRP1 and NLRP3 as biomarkers for the early detection of NMSC and targets for more personalized treatment strategies in improving patient outcomes.

## Methods

A comprehensive review of the literature was performed to examine the role of the NLRP1 and NLRP3 inflammasomes as tumour markers for NMSC. PubMed, Google Scholar and Web of Science were searched using terms including ‘NLRP1’, ‘NLRP3’, ‘inflammasomes’, ‘skin’, ‘non-melanoma skin cancer’, ‘NMSC’, ‘tumor markers’, ‘skin carcinogenesis’ and ‘inflammation’. The review focused on articles published between January 2000 and May 2024, to ensure inclusion of the most recent and relevant studies. Studies were selected based on the use of NLRP1 and NLRP3 inflammasomes as tumour markers, and if they addressed the expression, function and potential of these inflammasomes as tumour markers. Peer-reviewed sources, abstracts, conference papers and editorial comments were excluded to ensure scientific rigour and a focus on studies with adequate statistical power. This review highlights some of the key studies undertaken to understand the roles that the NLRP1 and NLRP3 inflammasomes play in NMSC development. The studies offered information on the expression levels and functional roles of these inflammasomes, together with possible implications as tumour markers.

## Results and discussion

### Role of NLRP1 and NLRP3 inflammasomes in ultraviolet-induced skin damage and inflammation

Inflammasomes represent important components of the innate immune system and are expressed in response to pathogen-associated molecular patterns (PAMPs), damage-associated molecular patterns (DAMPs) and cellular stress factors. At the centre of inflammasome activity are the production of NLRP1, CRD8, NLRP3, NLRP6, NLRC4/NAIP, AIM 2, pyrin and other sensors, each playing a distinct role in the recognition of specific stimuli and the inflammatory cascade. The process of inflammasome formation typically involves priming signals that prepare the cell and activation signals that directly trigger the assembly of multiprotein complexes, resulting in caspase-1 activation and subsequent cytokine production, particularly IL-1β and IL-18. Understanding the complex mechanisms by which these sensors operate in cellular contexts and their implications for immune modulation is helpful for finding new interventional treatments for inflammatory diseases and infection.^7^

**Figure 1:**
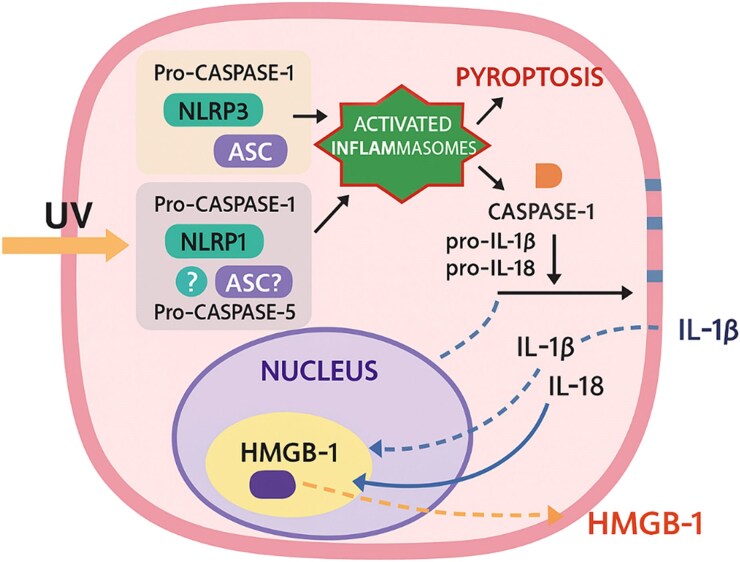
Activation mechanisms of NOD-like receptor family, pyrin domain containing 1 (NLRP1) and NOD-like receptor family, pyrin domain containing 3 (NLRP3) inflammasomes in human keratinocytes after ultraviolet (UV) radiation (UVR). Activation of the NLRP1 and NLRP3 inflammasomes can occur in human keratinocytes following UVR. The NLRP1 inflammasome complex includes caspase-1, apoptosis-associated speck-like protein containing a caspase recruitment domain (ASC; although ASC is not required for complex formation in murine cells) and NLRP1. The precise role of caspase-5 in NLRP1 inflammasome activation remains unclear. In contrast, the NLRP3 inflammasome, which is well-characterized among inflammasome complexes, comprises NLRP3, ASC and caspase-1. Active caspase-1 is necessary to process pro-interleukin (IL)-1β and pro-IL-18 into their mature forms, facilitating their secretion into the extracellular space. Additionally, the inflammasome is linked to the unconventional secretion of high mobility group box 1 (HMGB1). Active caspase-1 can induce cell pyroptosis, leading to membrane rupture and the release of HMGB1.

Ultraviolet (UV) radiation (UVR) is an important environmental hazard to the skin. It can directly damage the DNA of skin cells and trigger inflammation. This inflammation is marked by the activation of inflammasomes (NLRP1 and NLRP3) and stress pathways such as nuclear factor (NF)-κB, ultimately acting as a potent carcinogen by initiating and promoting carcinogenesis. UVR causes physiological and metabolic disruption, leading to the release of derived cellular metabolites (DAMPs), and noninflammatory inflammation or sterile inflammation (UVR-induced physiological and metabolic disruption leading to cellular changes without classical inflammatory symptoms such as redness, heat, swelling and pain). NOD-like receptors (NLRs) recognize specific DAMPs, including IL-1α and IL-33, which are potent danger signals that indicate potential loss of epidermal barrier integrity. A study has shown that UVB radiation can induce the secretion of IL-1β and IL-18 in human keratinocytes, resulting in an increase in inflammasomes.^4^ Although the NLRP1 and NLRP3 inflammasomes are involved in UV-induced inflammation, their specific roles differ, with NLRP1 potentially being the first responder to UV-induced DAMPs. Studies have shown that UVB-irradiated keratinocytes exhibit low levels of NLRP1, so a UV-induced mechanism of action of NLRP1 has been suggested.^2,3^ However, a conflicting study suggests that NLRP3, rather than NLRP1, may mediate UVB-induced IL-1β production in human keratinocytes. Despite these findings, the roles of NLRP3 and NLRP1 in UV-induced inflammation are poorly understood, highlighting the need for further investigation (Figure 1).^5^

Ciążyńska *et al.*^5^ highlighted the role of the NLRP1 and NLRP3 inflammasomes in skin carcinogenesis, focusing on their effects on tumour growth and immunity. In particular, NLRP1 was shown to participate in apoptotic pathways and cell cycle regulation.^5^ In a prospective observational study, Tan *et al.*^1^ found that low NLRP1 expression was significantly associated with an increased risk of metastasis and recurrence in patients with NMSC, suggesting it is a potential prognostic marker. Tan *et al.* included a cohort of 199 patients with cutaneous BCC (cBCC) and cutaneous SCC (cSCC), along with 199 blood samples from healthy individuals as a control group, with all patients being followed up for a duration of 1–3 years.^1^ Clinical stages were assessed using TNM staging, with TNM III–IV stages showing an association with lower NLRP1 levels. Statistical analysis methods included Kaplan–Meier analysis to evaluate NLRP1’s association with 1–3-year mortality and recurrence of NMSC, curvilinear regression to examine the relationship between NLRP1 and CEA/CYFRA21-1, and receiver operating characteristic curves to assess NLRP1 as a potential biomarker for lymph node metastasis, myometrial infiltration and prognosis. Subgroup analysis revealed that *NLRP1* expression was significantly higher in patients with cBCC compared with those with cSCC, while deceased patients, those with lymph node metastasis and those with myometrial infiltration exhibited significantly lower NLRP1 levels. Furthermore, lower NLRP1 levels were associated with higher frequencies of TNM III–IV stages, lymph node metastasis, myometrial infiltration, higher mortality and increased recurrence rates.^1^

Zhong *et al.*^3^ reported that germline mutations in *NLRP1* increased skin inflammation syndrome and cancer susceptibility, demonstrating its importance in skin cancer biology.^3^ This study investigated the role of inflammasome complexes as innate immune effectors that trigger inflammation in response to pathogen- and danger-­associated ­signals, focusing on two overlapping skin disorders: multiple self-healing palmoplantar carcinoma (MSPC) and familial keratosis lichenoides chronica (FKLC).^3^ The research highlighted NLRP1 as the most prominent inflammasome sensor in human skin, with all identified pathogenic mutations being gain-of-function alleles that enhance inflammasome activation. Mechanistically, these mutations disrupt the pyrin-like domains (PYD) and leucine-rich repeat (LRR) domains, promoting self-oligomerization of NLRP1 and resulting in spontaneous inflammasome activation and paracrine IL-1 signalling in primary keratinocytes, which cause skin inflammation and epidermal hyperplasia. The study established a genetic link between *NLRP1* mutations and skin inflammatory syndromes, including predisposition to skin cancer.^2^

Barry *et al.*^6^ reviewed recent studies on NLRP1, focusing on its activation mechanisms, gain-of-function human variants and its unique role in barrier cells such as keratinocytes and airway epithelial cells. By comparing NLRP1 with other inflammasomes like NLRP3, the review highlights NLRP1 as the predominant inflammasome in human barrier cells, primarily responding to cellular stress. Newly identified human-specific NLRP1 variants offer valuable insights into its biology and activation mechanisms, distinguishing it from other inflammasomes and establishing it as a key therapeutic target for inflammatory diseases. The findings suggest that NLRP1’s activation through gain-of-function variants presents a promising avenue for developing targeted therapies, emphasizing the need for further research into its mechanisms and clinical applications.^6^

Barry *et al.* also reported recent advances in *NLRP1* research, raising important questions about its mechanistic mechanisms and therapeutic potential.^6^ NALP1, also known as NLRP1, is a cellular sensor protein important in innate immunity and inflammation. It contains PYD, NACHT and LRRs, capable of recognizing a variety of stimuli such as microbial or cellular stress signals. Upon activation, NLRP1 forms an inflammasome complex with the adaptor protein ASC [apoptosis-associated speck-like protein containing a caspase recruitment domain (CARD)] and pro-caspase-1, which produces the activated form of the proinflammatory cytokines IL-1β and IL-18. This activity initiates an inflammatory response that is important for fighting infection and responding to cellular stress. Mutations in *NLRP1* have implications in autoimmune diseases, autoinflammatory syndromes, skin infections and some cancers, and have a role in immune system regulation and disease pathogenesis.^7^

CARD8, also known as caspase recruitment domain family member 8, is a protein crucial in regulating inflammatory responses and apoptosis. It features a CARD at its N-terminus, facilitating interactions with other CARD-containing proteins and caspases to modulate cellular processes. CARD8 is particularly involved in inflammatory signalling pathways, including those mediated by NF-κB and proinflammatory cytokines such as IL-1β and IL-18. Dysregulation or mutations in CARD8 have been linked to autoinflammatory syndromes like familial cold autoinflammatory syndrome and Muckle–Wells syndrome, where excessive inflammation results from aberrant innate immune activation. This underscores CARD8’s critical role in immune regulation and its implications in inflammatory diseases. Taabazuing *et al.*^8^ reviewed the specific activation mechanisms of the NLRP1 and CARD8 inflammasomes, suggesting a combined role in cancer. Fenini *et al.*^9^ extended the understanding of NLRP1 functions in the skin, affecting systemic immune responses.^10^

### NLRP1 inflammasome

NLRP1 is a protein characterized by five distinct domains that undergo self-processing. This self-processing occurs within the FIIND (function-to-find) domain, situated between the ZU5 (ZO-1 and UNC5) and UPA (UNC5, PIDD and ankyrins) subdomains. This process generates two functional forms: the inhibitory N-NLRP1 and the effector C-NLRP1. Germline mutations in *NLRP1*, known as stars, are associated with inflammatory skin conditions like MSPC and FKLC, predisposing individuals to SCC.

These mutations induce self-oligomerization and activation of NLRP1 through the release of C-NLRP1 from dipeptidyl peptidases DPP8/9 and the inhibitory N-NLRP1. UVB radiation activates NLRP1 via the ribotoxic stress response, triggering ZAKα kinase upon ribosome collision sensing. ZAKα then phosphorylates and activates stress-induced kinase p38, leading to phosphorylation of N-NLRP1 in the disordered linker region 1, resulting in its proteasomal degradation and subsequent release of C-NLRP1. This dual mechanism of activation suggests potential overlap in the pathways through which UVB radiation and ­disease-causing mutations contribute to SCC development.

**Figure 2:**
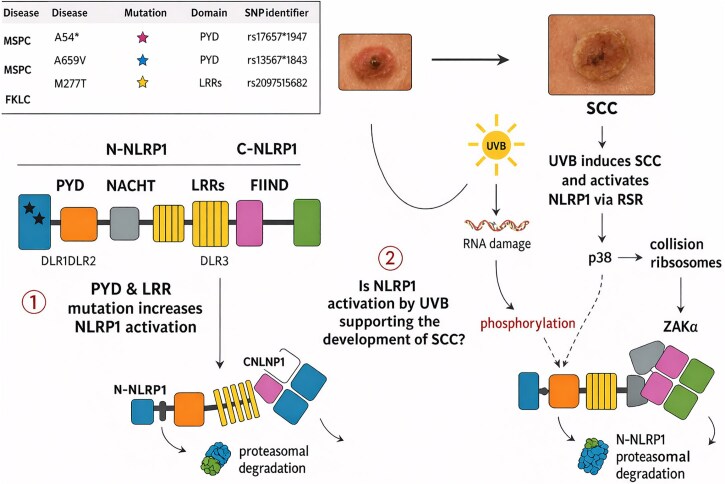
NOD-like receptor family, pyrin domain containing 1 (NLRP1), ultraviolet B (UVB) and skin cancer. C-NLRP1, effector NLRP1; FKLC, familial keratosis lichenoides carcinoma; LRR, leucine rich repeat; MSPC, multiple self-healing palmoplantar carcinoma; N-NLRP1, inhibitory NLRP1; RSR, ribotoxic stress response; SCC, squamous cell carcinoma; SNP, single nucleotide polymorphism.

Initially identified for its role in apoptosis, NLRP1 was subsequently recognized as the first inflammasome sensor, initially characterized in the monocytic cell line THP-1 but notably expressed at high levels in epithelial cells such as epidermal keratinocytes and the brain. The NLRP1 protein spans 1473 amino acids and includes domains such as the N-terminal PYD, NACHT domain, six LRRs, FIIND and carboxyterminal CARD. The FIIND domain, encompassing ZU5 and UPA subdomains, serves as a constitutive proteolytic self-activation domain where cleavage occurs specifically at Phe1212 at the ZU5-UPA border. The C-terminal segment (NLRP1-CT) functions as the effector fragment, remaining inhibited by interaction with the N-terminal inhibitory fragment (NLRP1-NT), with activation typically involving proteasomal degradation of NLRP1-NT (Figure 2).^9^

### NLRP3 inflammasome

NLRP3 is a 118-kDa cytosolic pattern recognition receptor (PRR) protein found in a variety of cells, including neutrophils, macrophages, microglia, lymphocytes, epithelial cells, osteoblasts, neurons and dendritic cells. Assembly of the NLRP3 inflammasome involves two major steps: priming and activation. Priming leads to increased transcription of NLRP3, proinflammatory cytokines (pro-IL-1β and pro-IL-18), and other molecules such as Toll-like receptors, NOD2, IL-1R or tumour necrosis factor receptor ligands in response to PAMPs and DAMPs such as lipopolysaccharide, tumour necrosis factor and IL-1β.^11^

Kummer *et al.*^4^ characterized the expression of NLRP1 and NLRP3 in different human tissues, suggesting that they play different roles in the inflammatory response.^4^ The study of Kummer *et al.* aimed to determine the distribution of NALP1 and NALP3 across various cells and tissues using monoclonal antibodies to identify their expression patterns.^4^ The findings revealed distinct profiles, with NALP1 broadly expressed by granulocytes, monocytes (weakly), dendritic cells, B and T cells, and most abundantly in T cells and Langerhans cells. NALP1 is also present in glandular epithelial structures such as the stomach, gut, lung, neurons and testis, primarily localized in the nucleus. In contrast, NALP3 shows a more restricted distribution, mainly found in nonkeratinizing epithelia of the oropharynx, oesophagus, ectocervix and the urothelial layer of the bladder, with predominant cytoplasmic localization. The study concludes that the presence of NALP3 in epithelial cells lining the oral and genital tracts facilitates the rapid sensing of invading pathogens, triggering an innate immune response. Additionally, the distinct subcellular localization of NALP1 (nuclear) and NALP3 (cytoplasmic) suggests functional differences that may influence their roles in immune signalling and inflammatory responses.^4^

The study by Sand *et al.*^2^ aimed to compare inflammasome activation between human and murine keratinocytes by stimulating both cell types with various inflammasome-activating stimuli, including UVB irradiation and cytokine priming, to assess their ability to produce inflammasome proteins and activate inflammasomes. The researchers measured the expression of inflammasome-related proteins (Nlrp1, Nlrp3, Aim2, Asc, pro-IL-1β) and the production of IL-1β and IL-18. The results showed that human keratinocytes effectively activated various inflammasomes and produced the proinflammatory cytokines IL-1β and IL-18 in response to stimuli, while murine keratinocytes displayed very low or undetectable levels of inflammasome proteins and did not activate inflammasomes even after cytokine priming. Although murine keratinocytes produced caspase-1 and pro-IL-18, inflammasome activation was absent, and UVB-induced inflammation and neutrophil recruitment depended on IL-1β and caspase-1, with pro-IL-1β expression detected only in immune cells and not keratinocytes. The study concludes that human keratinocytes have higher immunological competence than murine keratinocytes, as demonstrated by stress-induced IL-1β secretion mediated by inflammasomes. This highlights a fundamental difference in immune function, where human keratinocytes can perform immune roles that are instead carried out by professional immune cells in murine skin.^3^ The study by Nie *et al.*^12^ investigated the effects of 5-aminolaevulinic acid–photodynamic therapy (ALA-PDT) on cSCC using a mouse model and various cell lines.^12^ Female SKH-1 hairless mice were induced with cSCC through UV irradiation, and murine SCC cells and murine cancer-associated fibroblasts (mCAFs) were maintained in appropriate culture conditions. The study utilized gene chips, quantitative polymerase chain reaction (qPCR), enzyme-linked immunosorbent assay (ELISA), implanted tumour models, Western blotting and immunohistochemical studies to assess the role of IL-1β production and its mechanism in response to ALA-PDT. Microarray analysis of SCC tumours before and after ALA-PDT revealed increased IL-1R1 and IL-1β expression, mainly in mesenchymal cells. *In vivo* experiments showed that inhibiting IL-1β or caspase-1 significantly reduced ALA-PDT’s effectiveness in suppressing tumour growth. Further analysis indicated that ALA-PDT activated the NLRP3 inflammasome and p-p65/p65 pathway in CAFs, contributing to IL-1β production. ELISA and Western blotting confirmed the involvement of NLRP3, ASC, caspase-1 and NF-κB pathways. The results suggest that ALA-PDT stimulates a local acute inflammatory response through IL-1β production in CAFs, enhancing its therapeutic effect on cSCC.^12^

The study by Miao *et al.* focuses on pyroptosis, a form of programmed cell death triggered by caspase-1 activation through various inflammasomes, resulting in lysis of the affected cell and inflammatory cytokine secretion (IL-1β and IL-18).^13^ Unlike apoptosis, which is noninflammatory, pyroptosis is inflammatory and primarily occurs in macrophages and dendritic cells.^13^ The study highlights the role of different inflammasomes (NLRC4, NLRP3, AIM2) and the importance of the ASC focus in caspase-1 signalling. Pyroptosis serves as a potent innate immune mechanism to clear intracellular pathogens by releasing them from infected cells for phagocytic clearance by neutrophils. The findings emphasize the critical role of pyroptosis in host defence mechanisms and its potential as a therapeutic target in infectious diseases.^12,13^

Ahmad *et al.*^14^ described the role of NLRP3 inflammasome activation in BCC and the involvement of Ca^2+^ mobilization in this process. Human primary BCC samples were obtained from sun-exposed skin sites in 10 patients postsurgery, and HaCaT keratinocytes were used to examine the molecular mechanisms of NLRP3 activation following UVB exposure.^14^ The research demonstrated that UVB exposure blocks Ca^2+^ mobilization by downregulating the expression of sarcoplasmic/endoplasmic reticulum Ca^2+^-ATPase (SERCA2), a crucial component of store-operated Ca^2+^ entry, leading to the activation of the NLRP3 inflammasome. This activation results in caspase-1 processing of pro-IL-1β into its active form, IL-1β, suggesting that NLRP3 plays a role in the inflammatory response associated with BCC progression. Statistical analysis was performed using the two-tailed Student’s *t*-test for immunostaining, qPCR and Western blot analysis, without employing advanced statistical methods. The findings indicate that UVB-induced NLRP3 inflammasome activation through Ca^2+^ mobilization disruption could be a potential target for therapeutic intervention in BCCs.^14^

A comprehensive review by Chavarría-Smith and Vance discusses recent findings on the activation of the NLRP1 inflammasome, focusing on its unique mechanisms of activation by various stimuli such as *Bacillus anthracis* lethal toxin, *Toxoplasma gondii*, muramyl dipeptide and host intracellular ATP depletion.^15^ It highlights NLRP1’s role in pathogen recognition and resistance during infection, along with its regulation by host and viral proteins. The review reveals that NLRP1 acts as a cytosolic sensor forming an inflammasome upon activation by microbial and endogenous signals, leading to caspase-1 processing, maturation of cytokines IL-1β and IL-18, and induction of pyroptosis, a lytic cell death essential for pathogen clearance. Unlike other inflammasomes such as NAIP/NLRC4, NLRP1 exhibits distinct activation mechanisms, especially in response to intracellular ATP depletion and specific pathogen-associated factors.^15^

Zhai *et al.*^16^ investigated the role of the NLRP1 inflammasome in acquired drug resistance to temozolomide (TMZ) in melanoma, focusing on O^6^-methylguanine-DNA methyltransferase (MGMT)-low human melanoma cell lines (1205Lu and HS294T) that are initially sensitive to TMZ but develop resistance through MGMT-independent mechanisms. The cells were treated with TMZ for over 2 months, during which researchers assessed changes in *NLRP1* expression, NLRP inflammasome activation, IL-1β secretion and NF-κB activity.^16^ The results demonstrated that prolonged TMZ exposure led to MGMT upregulation and acquired resistance; however, this resistance was independent of MGMT and instead associated with increased *NLRP1* expression, inflammasome activation, IL-1β secretion and NF-κB activity. Blocking IL-1R signalling with an IL-1R antagonist effectively reduced TMZ-resistant 1205Lu tumour growth *in vivo*. The findings indicate that NLRP1 acts as both a melanoma tumour promoter and an apoptosis suppressor, contributing to the development of acquired drug resistance. The study is the first to establish a direct link between NLRP1 and acquired drug resistance, highlighting it as a potential therapeutic target for drug-­resistant melanoma. Additionally, the results suggest that drug-tolerant cancer cells may become cross-tolerant to other classes of cancer drugs, indicating a shared mechanism involving NLRP1 in resistance to various therapies.^16^

**Figure 3:**
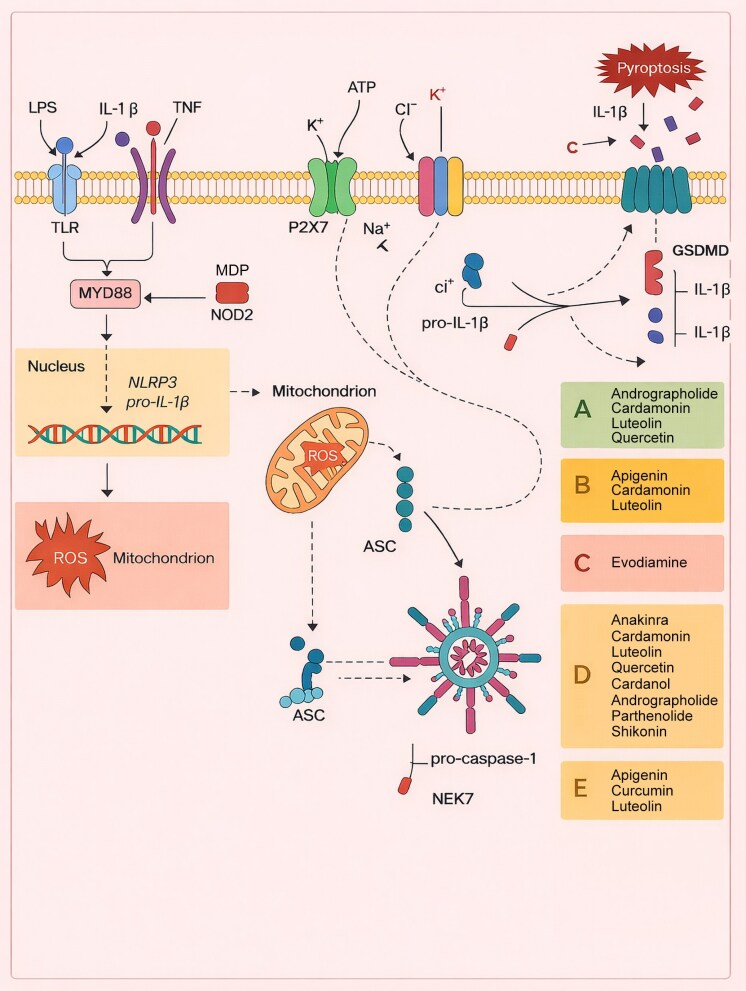
The NOD-like receptor family, pyrin domain containing 3 (NLRP3) inflammasome pathway and its inhibitors. ASC, apoptosis-associated speck-like protein containing a caspase recruitment domain; GSDMD, gasdermin D; IL, interleukin; LPS, lipopolysaccharide; MDP, muramyl dipeptide; NOD2, nucleotide-binding oligomerization domain 2; ROS, reactive oxygen species; TLR, Toll-like receptor; TNF, tumour necrosis factor.

The association between the regulated NLRP3 inflammasome and various human diseases highlights the potential of NLRP3 inhibitors in therapeutic approaches. Recent studies have identified several inhibitors of the NLRP3 inflammasome, some of which are currently in clinical trials. In this study, a system–activity relationship analysis of the relevant mechanisms of inhibition developed with direct and indirect inhibitors targeting the NLRP3 inflammasome was proposed (Figure 3).^17^

### NLRP1 as a tumour marker in nonmelanoma skin cancer

NLRP1 seems to be one of the leading contributors to the genesis and evolution of NMSCs. Most likely, it modulates inflammation activation that has an impact on tumorigenesis. It has been shown that the inflammatory pathway mediated by NLRP1 is potently inhibited. The antitumour efficacy of NLRP1 in SCC supports the idea of potential tumour suppressor activity of NLRP1, contributing to uncontrolled cell proliferation and tumour growth. Tan *et al.*^1^ found that low expression levels of NLRP1 were able to induce higher metastasis and recurrence in patients with NMSC, thus acting as a tumour suppressor.^1^ These findings support of NLRP1 expression as a potential prognostic marker in the clinical setting.

### Genetic variants of *NLRP1* confer individual susceptibility to skin cancer

Ciłzyńska *et al*.^5^ observed that variants of *NLRP1* can alter the response to environmental carcinogens and inflammatory stimuli. This also points towards the role of inflammation in the pathogenesis of NMSCs.^5^ There is an association of *NLRP1* polymorphisms with melanoma risk. This is particularly true in populations where there is a high incidence of UV exposure. Taken together, these findings again point to the potential of NLRP1 not only as a biomarker, but also as a target for therapy as its loss of expression could be restored to dampen tumour development.

### Potential synergy between NLRP1 deficiency and p53 mutations in tumour progression

NLRP1 is a PRR belonging to the NLR family, which plays a crucial role in the innate immune system. It functions by forming an inflammasome complex that leads to the activation of caspase-1 and subsequent maturation of pro­inflammatory cytokines such as IL-1β and IL-18.^18^ NLRP1 is involved in immune surveillance and inflammation-induced apoptosis, which are critical for preventing tumorigenesis. Deficiency or dysfunction of NLRP1 has been associated with impaired immune responses and a favourable environment for tumour growth.^19^ The p53 protein, often referred to as the ‘guardian of the genome,’ is one of the most well-known tumour suppressors. It plays a pivotal role in regulating cell cycle arrest, DNA repair, apoptosis and senescence in response to cellular stress or DNA damage.^20^ Mutations in p53 are common across various cancers, occurring in approximately 50% of all human tumours, resulting in loss of function or the acquisition of oncogenic properties.^21^ Loss of p53 function leads to increased genomic instability, inhibition of apoptosis and uncontrolled cellular proliferation.

### Mechanisms of potential synergy

Impaired apoptosis and inflammasome activation are likely significant contributors to the potential synergy between NLRP1 deficiency and p53 mutations. NLRP1 deficiency reduces caspase-1 activation, thereby decreasing pyroptosis and the release of proinflammatory cytokines.^22^ Meanwhile, p53 mutations compromise the intrinsic and extrinsic apoptosis pathways, further promoting the survival of damaged cells that would typically undergo programmed cell death.^23^ Additionally, disruption of immune surveillance is another potential mechanism that drives this synergy. NLRP1 deficiency impairs immune cell recruitment and alters the polarization of immune responses, reducing the efficiency of antitumour immunity.^18^ Concurrently, p53 mutations contribute to immune evasion by upregulating immune checkpoint proteins such as programmed cell death ligand 1 (PD-L1), further promoting immune suppression.^24^

The combination of NLRP1 deficiency and p53 mutation can also enhance genomic instability and mutagenesis. Loss of p53 function leads to defective DNA repair mechanisms and the accumulation of mutations.^21^ NLRP1 deficiency may exacerbate this process by disrupting inflammation-driven DNA damage repair pathways, leading to accelerated tumour progression.

Furthermore, the synergistic effects of NLRP1 deficiency and p53 mutations extend to enhanced proliferative and survival signals. Loss of p53 function allows uncontrolled cell cycle progression, even in the presence of DNA damage. Simultaneously, NLRP1 deficiency promotes ­prosurvival pathways, creating a more favourable environment for tumour growth and metastasis.^18^

### Clinical relevance and implications

Numerous studies have demonstrated that NLRP1 ­downregulation is associated with poor prognosis in various cancers, including colorectal cancer, melanoma and lung cancer. Similarly, mutations in *TP53* are strongly correlated with more aggressive tumour behaviour and poor treatment outcomes.^21^ The coexistence of NLRP1 deficiency and *TP53* mutations may represent a particularly aggressive cancer phenotype with enhanced resistance to conventional therapies.

### Potential therapeutic approaches

Targeting the NLRP1 and p53 pathways may provide novel therapeutic opportunities. Small-molecule agonists to restore NLRP1 inflammasome function could enhance immune-mediated tumour suppression. However, restoring wildtype p53 function through gene therapy or small molecules like PRIMA-1 and APR-246 has shown promise in preclinical studies.^25^ Combination therapies targeting both pathways simultaneously could improve treatment efficacy and reduce tumour progression.

Immune checkpoint inhibition, particularly targeting the programmed cell death protein 1/PD-L1 pathway, may also be beneficial in cancers where both NLRP1 deficiency and *TP53* mutations are present. Moreover, exploring synthetic lethality approaches that target vulnerabilities created by these deficiencies may open new avenues for cancer therapy.

Future research should investigate the co-expression patterns of *NLRP1* and *TP53* across various cancer types. Functional studies exploring the impact of NLRP1 activation in p53-deficient cancers could provide insights into potential therapeutic strategies. Developing mouse models with combined NLRP1 deficiency and *TP53* mutations would allow further understanding of their synergistic effects on tumour progression.^24^

### NLRP3 as a tumour marker in nonmelanoma skin cancer

The involvement of NLRP3 in tumorigenesis has been investigated mainly regarding its role in inflammatory processes. Ahmad *et al.*^14^ investigated the role of the NLRP3 inflammasome in BCC activation induced by UVB radiation.^14^ Human primary BCC samples were collected from sun-exposed skin sites from 10 patients postsurgery, and HaCaT keratinocyte cells were exposed to UVB radiation (100 mJ cm^–^²) to explore the molecular mechanisms underlying NLRP3 activation. Various assays, including quantitative real-time PCR, Western blot, immunofluorescence and ELISA, were employed to assess the expression of NLRP3, caspase-1, SERCA2 and IL-1β. Intracellular calcium levels were measured using Fura-2 AM fluorescence, with treatments involving thapsigargin, extracellular calcium and BAPTA-AM to evaluate the dependence of NLRP3 activation on calcium signalling. Statistical analysis was conducted using the two-tailed Student’s *t*-test, with significance defined as *P* < 0.05. The results showed that NLRP3 expression was significantly elevated in BCC samples compared with normal skin, and UVB exposure increased intracellular calcium levels, activating the NLRP3 inflammasome, resulting in enhanced caspase-1 activation and IL-1β secretion. The study further demonstrated that UVB radiation downregulated SERCA2, a critical component of store-operated Ca^2+^ entry, contributing to elevated intracellular calcium levels. Blocking calcium signalling using BAPTA-AM significantly reduced IL-1β production, confirming the ­calcium-dependent activation of NLRP3. The findings suggest that UVB-induced NLRP3 inflammasome activation occurs through the disruption of calcium homeostasis via SERCA2 downregulation, presenting a potential therapeutic target for preventing or treating UVB-induced BCC progression.^14^

NLRP3 has been shown to interrelate with inflammatory cytokines, especially IL-1β and IL-18, in contributing to the tumour microenvironment (TME) of skin cancers, tumour growth and metastasis. More importantly, Nie *et al.*^12^ investigated the role of NLRP3 inflammasome-mediated IL-1β production in CAFs in enhancing the effectiveness of ALA-PDT in treating cSCC.^12^ Using a UV-induced cSCC model in female SKH-1 hairless mice, researchers performed inflammatory cytokine screening with gene chips, qPCR, ELISA and Western blotting to evaluate IL-1β expression. Implanted tumour models treated with anti-IL-1β monoclonal antibody or caspase-1 inhibitors were used to assess the impact of IL-1β inhibition on ALA-PDT efficacy. Microarray analysis, immunohistochemistry and statistical analysis (Student’s *t*-test) were conducted to study gene expression changes and therapeutic outcomes. The results demonstrated that ALA-PDT significantly increased IL-1R1 and IL-1β expression, particularly in mesenchymal cells, suggesting that CAFs are the primary producers of IL-1β post-treatment. Blocking IL-1β activity or caspase-1 *in vivo* reduced ALA-PDT efficacy, indicating the essential role of IL-1β in enhancing antitumour effects. Additionally, NLRP3 inflammasome activation was confirmed by increased expression of NLRP3, ASC, caspase-1 and p-p65/p65 in CAFs, suggesting that the NF-κB pathway mediates IL-1β production. The study concluded that ALA-PDT enhances IL-1β production via NLRP3 inflammasome activation in CAFs, contributing to improved antitumour immunity against cSCC, and highlighted that targeting NLRP3 or IL-1β could further enhance the therapeutic efficacy of ALA-PDT.^12^

These findings support the emerging concept that NLRP3 can be both tumour-suppressive and tumour-promoting, depending on the TME. Recent studies have identified NLRP3 as a potential biomarker; its expression levels have been said to indicate both the stage and prognosis of the tumour in NMSC. Schroder *et al.*^18^ discussed how inflammasomes, particularly NLRP3, NLRP6 and NLRP12, regulate intestinal mucosal immune responses, focusing on their roles in maintaining homeostasis, responding to enteric infections, managing autoinflammation and influencing tumorigenesis. Using various experimental models, including knockout mice, bone marrow chimeric mice, dextran sulfate sodium (DSS) colitis models and azoxymethane-chronic DSS models, researchers employed techniques such as ELISA, qPCR, immunohistochemistry and microbiota analysis to examine disease severity and microbial changes. The results demonstrated that NLRP3 in haematopoietic cells protects against colitis-associated tumorigenesis via IL-18 secretion, NLRP6 regulates microbial ecology and prevents autoinflammation in non­haematopoietic cells, and NLRP12 acts as a negative regulator of NF-κB signalling, with its deficiency increasing inflammation and colorectal cancer susceptibility. Dysregulated inflammasome activity leads to aberrant microbial colonization, promoting inflammation and tumorigenesis. The study concludes that targeting specific inflammasomes or their pathways could be a therapeutic approach for treating inflammatory bowel disease and related colorectal cancer.

The study by Ciążyńska *et al.*^5^ reviewed the activation and regulation of the NLRP1 and NLRP3 inflammasomes in skin carcinogenesis, focusing on their roles in response to UVR. Researchers compared how the NLRP1 and NLRP3 inflammasomes detect cellular stress and trigger inflammatory responses, particularly examining UVR-induced responses in keratinocytes and fibroblasts. The findings revealed that NLRP3 inflammasome activation in keratinocytes exposed to UVB radiation leads to the secretion of IL-1β and IL-18, contributing to inflammatory responses and potential tumorigenesis. In contrast, NLRP1 activation is more closely associated with the suppression of apoptosis and tumour progression in melanoma. Both inflammasomes are linked to oxidative stress, DNA damage and inflammatory cytokine production, which contribute to skin carcinogenesis. The study concluded that NLRP3 promotes inflammation through cytokine release, while NLRP1 contributes to tumour progression by inhibiting apoptosis. Targeting these inflammasomes may provide therapeutic strategies for treating skin cancers associated with UVR-induced damage.^5^

### NLRP3 inflammasome regulatory networks in the tumour microenvironment

The NLRP3 inflammasome plays a crucial role in regulating immune responses within various TMEs. It is composed of NLRP3, ASC and pro-caspase-1. Upon activation, the inflammasome cleaves pro-caspase-1 into active caspase-1, leading to the maturation of proinflammatory cytokines such as IL-1β and IL-18, which drive inflammatory responses and pyroptosis. The activation of the NLRP3 inflammasome is influenced by several signalling pathways, including transforming growth factor (TGF)-β signalling and reactive oxygen species (ROS)-dependent activation.

TGF-β is a key immunosuppressive cytokine in the TME that promotes tumour progression by inhibiting antitumour immune responses and enhancing epithelial-to-­mesenchymal transition. TGF-β signalling generally acts to suppress NLRP3 inflammasome activation, thus contributing to an immunosuppressive environment conducive to tumour growth.^26^ The mechanism involves direct inhibition of *NLRP3* transcription, enhancing the expression of anti-­inflammatory cytokines such as IL-10, and interacting with Smad signalling pathways to modulate transcriptional control over inflammasome-related genes.^27^

In contrast, ROS are byproducts of cellular metabolism that are increased under hypoxic and stressful conditions within the TME. ROS act as potent activators of the NLRP3 inflammasome through oxidative stress mechanisms, promoting inflammation and, in some contexts, supporting tumour growth and metastasis.^28^ ROS disrupt mitochondrial integrity, leading to the release of mitochondrial DNA, which serves as a danger signal activating NLRP3. ROS can also modify redox-sensitive proteins that interact with NLRP3, promoting its activation. In some tumours, elevated ROS levels enhance the NLRP3 inflammasome, thereby promoting inflammation that supports tumour growth and metastasis.^27,28^

The crosstalk between TGF-β signalling and ROS-dependent NLRP3 activation is complex. While TGF-β signalling often inhibits NLRP3 activation, it can also influence ROS production, thereby enhancing oxidative stress and indirectly affecting NLRP3 activation through downstream pathways.^29^ Furthermore, the specific responses of the NLRP3 inflammasome within different TMEs vary across tumour types. For example, NLRP3 activation can enhance antitumour immunity in some cancers, whereas in others it promotes a protumorigenic inflammatory environment. Targeting NLRP3 activation, considering the context of TGF-β and ROS signalling, offers potential therapeutic strategies to modulate the immune landscape of the TME towards a more favourable antitumour response.^28,29^ As has been mentioned, the NLRP3 inflammasome plays a complex role in cancer progression, exhibiting tumour-suppressive effects during early stages and metastasis-promoting functions in advanced stages.

### Tumour-suppressive effects in early cancer stages

In the initial phases of cancer development, NLRP3 activation contributes to tumour suppression through several mechanisms. Activation of the NLRP3 inflammasome leads to the production of IL-18, a cytokine that enhances epithelial integrity and promotes tissue repair, thereby reducing tumour formation. This phenomenon has been particularly well studied in colitis-associated colorectal cancer models, where NLRP3-mediated IL-18 production plays a crucial role in maintaining gut homeostasis and preventing early tumorigenesis.^30,31^

While the direct effects of NLRP3 activation in skin cancers, particularly BCC and SCC, are less established, parallels can be drawn from other epithelial cancers. The protective role of NLRP3 may involve enhancing immune responses and maintaining skin barrier integrity, thereby reducing the risk of early-stage tumorigenesis.

### Metastatis-promoting roles in advanced cancer stages

Conversely, in advanced stages of cancer, NLRP3 activation has been associated with promoting metastasis. Studies have demonstrated that NLRP3 activation within tumour-associated macrophages (TAMs) enhances the metastatic potential of cancer cells by promoting a pro­inflammatory TME. In epithelial ovarian cancer, for example, NLRP3 activation contributes to metastasis through the production of inflammatory cytokines that support tumour growth and invasion.^32^ Moreover, in SCC, NLRP3 activation has been linked to the promotion of carcinogenesis by fostering a proinflammatory environment that supports tumour progression. Specifically, NLRP3 activation leads to the production of proinflammatory cytokines such as IL-1β and IL-18, which can enhance cancer stem cell self-renewal and tumour development.^11^ This inflammatory response creates conditions favourable for cancer cell migration, invasion and metastasis.

The contrasting roles of NLRP3 in cancer progression underscore its dualistic nature. While it functions as a tumour suppressor during early stages by enhancing immune responses and promoting tissue integrity, chronic activation of NLRP3 in advanced stages facilitates metastasis through mechanisms that promote tumour cell proliferation, invasion and immune evasion. Understanding these molecular mechanisms may offer new therapeutic targets for treating advanced skin cancers, particularly SCC.

## Clinical relevance and future directions

### Translational medicine perspective

Targeting inflammasomes like NLRP3 and NLRP1 presents promising avenues for skin cancer therapies, particularly in BCC and SCC. In SCC, NLRP3 activation has been linked to the promotion of carcinogenesis through a proinflammatory environment that supports tumour progression. Additionally, NLRP1, which is highly expressed in keratinocytes, may play a role in skin cancer development and progression. Modulating inflammasome activity using small-molecule inhibitors such as MCC950, which targets NLRP3, could offer therapeutic benefits by mitigating inflammatory responses and reducing cancer stem cell self-renewal and tumour progression.^33^

### Cross-pathway synergy analysis: NLRP3 activation and PD-L1 expression

The NLRP3 inflammasome plays a pivotal role in the innate immune system by detecting cellular stress and damage, leading to the activation of inflammatory responses through the maturation and secretion of cytokines such as IL-1β and IL-18. Recent studies have shown that NLRP3 activation can significantly influence the expression of PD-L1, a critical molecule involved in tumour immune evasion.^34^ This interaction primarily occurs through the NF-κB signalling pathway, a crucial regulator of immune responses and inflammation. Activation of NLRP3 can stimulate NF-κB signalling, leading to the transcription of inflammatory genes, including *CD274*, which enhances immune suppression in cancer cells.^35^

### Inflammatory cytokine production and immune suppression

The NLRP3 inflammasome promotes the release of the proinflammatory cytokines IL-1β and IL-18, contributing to a proinflammatory TME that further enhances PD-L1 expression. The inflammatory environment resulting from NLRP3 activation also affects various immune cells within the TME, such as TAMs and myeloid-derived suppressor cells, which can upregulate PD-L1 expression and contribute to the suppression of antitumour immune responses.^36^ Moreover, the connection between NLRP3 activation and PD-L1 upregulation suggests that inflammasome-mediated PD-L1 expression may contribute to resistance against PD-1 blockade therapies, complicating the efficacy of immunotherapy.

### Therapeutic implications and biomarker potential

Given the interplay between NLRP3 activation and PD-L1 expression, targeting the NLRP3 inflammasome alongside PD-1/PD-L1 checkpoint inhibitors presents a promising therapeutic strategy. For instance, the use of NLRP3 inhibitors in combination with anti-PD-1 therapies has demonstrated the potential to reduce tumour growth and enhance immune responses. Furthermore, assessing NLRP3 activation status and PD-L1 expression levels in tumours could serve as biomarkers to predict patient responses to immunotherapies and guide personalized treatment strategies.

### Clinical trials and preclinical advances

Phase I/II clinical trials of MCC950, an NLRP3 inhibitor, are currently being investigated for their therapeutic potential in skin cancers, including SCC. Early results demonstrate that MCC950 effectively inhibits inflammasome activation, reducing IL-1β and IL-18 production, thereby limiting cancer cell proliferation and inflammation.^36^ Additionally, preclinical studies involving *NLRP1* gene-editing approaches, particularly using CRISPR/Cas9, have demonstrated promising results in reducing *NLRP1* expression in keratinocytes, resulting in decreased tumour growth and enhanced sensitivity to standard therapies.^37^ Growing evidence for the involvement of NLRP1 and NLRP3 in the pathogenesis of NMSC points toward a need for clinical studies that could translate into practical applications. Future studies are necessary to focus on targeted therapies that modulate these inflammasome activities. These may include clinical trials that will validate whether inhibitors of NLRP1 or NLRP3 can be combined with current treatments to form novel strategies that improve outcomes. Given the complexity of inflammasome biology, investigations of combination therapies with such targets together with immunotherapies also likely hold considerable promise. Furthermore, diagnostic tools that measure the expression levels of NLRP1 and NLRP3 in tumour biopsies might be very useful for personalized treatment. Further studies of inflammasomes in other epithelial cancers might broaden their applicability. The role of NLRP1 and NLRP3 in inflammation and cancer biology predicts an impact beyond NMSC. Such studies on their functions in lung, breast or gastrointestinal cancers would provide clues as to their universal mechanisms of action and therapeutic potential. Although the current view of NLRP1 and NLRP3 in NMSC is evolving, the potential application for these inflammasomes toward next-generation therapeutic development is tremendous. Prioritizing studies that bridge basic findings in the laboratory to the clinic will enable the development of novel strategies against skin cancer and perhaps other epithelial tumours.

### Application of CRISPR/Cas9 in squamous cell carcinoma and basal cell carcinoma

CRISPR/Cas9 gene-editing technology has emerged as a powerful tool for understanding the genetic basis of various cancers, including SCC and BCC. Its utility in identifying genetic vulnerabilities and potential therapeutic targets has been demonstrated in several studies, particularly focusing on SCC.

#### CRISPR/Cas9 and squamous cell carcinoma

Most of the current research has concentrated on oral SCC (oSCC), a subtype of SCC. Researchers have utilized genome-wide CRISPR/Cas9 screens to uncover genetic dependencies that are crucial for oSCC survival. In a study conducted on 21 oSCC cell lines derived mainly from Asian patients, the essentiality of *YAP1* and WWTR1 was validated, revealing mutually exclusive dependencies in different oSCC subsets.^38^ This study provides important insights into subtype-specific therapeutic targeting. Another notable study used CRISPR/Cas9 in the human oSCC cell line. The results demonstrated a significant reduction in the migration and proliferation abilities of these cells, indicating the role of integrin beta-6 subunit in oSCC progression and its potential as a therapeutic target. Furthermore, CRISPR/Cas9 has been applied to target *MMP9* in cutaneous SCC cell lines. Transfection with *MMP9*-specific guide RNAs resulted in the downregulation of *MMP9* expression, suggesting a possible anticancer strategy through gene suppression.^37,39^

#### CRISPR/Cas9 and basal cell carcinoma

Compared with SCC, the application of CRISPR/Cas9 in BCC research remains limited. However, studies have suggested that CRISPR/Cas9 technology could be beneficial in modelling cancer predisposition syndromes related to BCC, such as Gorlin syndrome. Through the introduction of specific genetic mutations into human cell lines, researchers can gain insights into BCC pathogenesis and potentially develop targeted therapies.^40^ Overall, CRISPR/Cas9 technology has provided valuable insights into the genetic mechanisms underlying SCC, especially oSCC, and holds promise for BCC research. Its applications are expanding, although more studies are needed to fully realize its potential in BCC treatment and prevention.

## Conclusion

The therapeutic potential of targeting the NLRP1 and NLRP3 inflammasomes in NMSCs is increasingly evident. NLRP1 shows promise as a prognostic marker, especially in SCC, where its tumour-suppressive role is linked to controlling inflammation and preventing uncontrolled cell proliferation. Genetic variants affecting NLRP1 function highlight its importance as a therapeutic target, with strategies aimed at restoring its function offering a potential approach to mitigate tumour progression. NLRP3 has a dual role, depending on cancer stage. While early-stage activation of NLRP3 enhances antitumour immunity, its chronic activation in advanced cancer stages contributes to metastasis and tumour growth. Therefore, inhibiting NLRP3-mediated inflammation in the TME represents a valuable therapeutic strategy.

Future research should focus on developing combination therapies targeting inflammasome pathways and integrating them with existing immunotherapies. This may involve approaches such as CRISPR/Cas9-mediated modulation of inflammasome activity or the application of inflammasome inhibitors like MCC950. Clinical trials aimed at assessing the efficacy of these therapies, particularly in conjunction with immune checkpoint inhibitors, are necessary to determine their effectiveness in improving patient outcomes.

Furthermore, diagnostic tools that evaluate NLRP1 and NLRP3 expression levels in tumour biopsies could enhance personalized treatment approaches. Investigating the applicability of these inflammasomes in other epithelial cancers could broaden their therapeutic relevance.

## Data Availability

The data underlying this article will be shared on reasonable request to the corresponding author.
